# Cycling in the dark – the impact of Standard Time and Daylight Saving Time on bicycle ridership

**DOI:** 10.1093/pnasnexus/pgab006

**Published:** 2022-03-02

**Authors:** Jan Wessel

**Affiliations:** Institute of Transport Economics, University of Münster, Am Stadtgraben 9, 48143 Münster, Germany

**Keywords:** cycling, Daylight Saving Time, sunrise and sunset, bike ridership, automated counting stations

## Abstract

The European Union is in the process of abolishing the bi-annual clock change. Against this backdrop, we analyze how daylight and twilight affect the sustainable transport mode of cycling, and find that better daylight conditions generally lead to higher levels of cycling. The extent of this effect depends on the type of traffic and the time of day. An all-year implementation of Daylight Saving Time would then lead to an increase in overall cycling levels of around 3.14 %–3.37 %, compared to an all-year Standard Time. This would imply an increase of around 1.27–1.36 billion cycled kilometers per year in Germany alone. Additionally, we provide monetary estimates for the external effects of such changes in cycling levels.

Significance Statement Cycling impacts positively on people’s health, and entails benefits for both society and environment. Increasing the number of cyclists is, therefore, considered an important means of promoting more sustainable mobility. We find that bicycle use is significantly affected by the level of daylight and twilight. This has important consequences for the debate on abolishing the bi-annual clock change, which was decided in the EU in 2018, but has not yet been implemented. Using German data, we show that an all-year Daylight Saving Time would lead to higher levels of cycling than an all-year Standard Time, or the current time regime with a bi-annual clock change. We also provide lower-bound estimates for the economic consequences of implementing all-year time regimes.

## Introduction

In 2018, the European Union (EU) conducted an online survey among its inhabitants with regard to the acceptance of Daylight Saving Time (DST). A total of 4.6 million people participated in this online survey, and the results showed that 84 % were in favor of stopping the bi-annual clock change (1). Despite the initially stated desire to quickly abolish the bi-annual clock change, for example by the then-President of the EU Jean-Claude Juncker, the bi-annual clock change has still not been abolished, and there appears to be no clear plan as to when—or even if—it should indeed be abolished. A reason for this is that an abolishment of the bi-annual clock change would be associated with several points of contention, as each country would then have to decide on a time regime that is valid for the whole year. On the one hand, establishing the same time regime for each country could be problematic for those on the geographic fringes of the EU. On the other hand, the implementation of different time regimes within the EU would lead to a patchwork of different time regimes, thereby complicating daily business. Even if we suppose that each country could decide on an all-year time regime on its own, without factoring in the ramifications for other EU partner countries, one very important question remains: which time regime should be implemented?

This question can of course be analyzed against the backdrop of social, economical, health-related, or other aspects (2–5). With respect to traffic, there are various papers that analyze the impact of the clock change on traffic crashes (6,7). Most recently, Bünnings and Schiele (8) have shown that darkness is an important factor for crashes, and that an all-year implementation of DST would significantly decrease road crashes in Great Britain, and thereby also reduce social costs.

Light conditions also significantly affect the attractiveness of cycling. As Uttley and Fotios (9) point out, good light conditions can help cyclists to detect and avoid hazardous obstacles (10), increase their perceived safety of the environment (11), and increase their perceived self-visibility and the corresponding level of safety (12). Hence, changes in light conditions due to clock changes could lead to changes in bicycle ridership, and thereby help to tackle environmental problems associated with the transport sector. In the transport sector, the promotion of cycling is considered an important cornerstone for reducing emissions and congestion (13,14).

The literature on the impact of sun positions and the accompanying variations in light conditions on actual bicycle ridership is, however, rather scarce. Uttley and Fotios (9) exploit the bi-annual clock change by comparing bicycle ridership between hourly observations shortly before and shortly after the clock change, which entails a significant and immediate change in light conditions. They controlled for ridership changes between hours that had no such differences in light conditions. Their results indicate a positive impact of more daylight on bicycle ridership. Fotios et al. (15) use hourly observations from the whole year, and compare bicycle ridership between an hour that always has daylight, an hour that is always dark, and an hour with changing light conditions. They also find a positive effect of daylight on bicycle ridership. In addition, Goodman et al. (16) show that a later sunset due to DST slightly increases children’s daily physical activity.

**Fig. 1:**
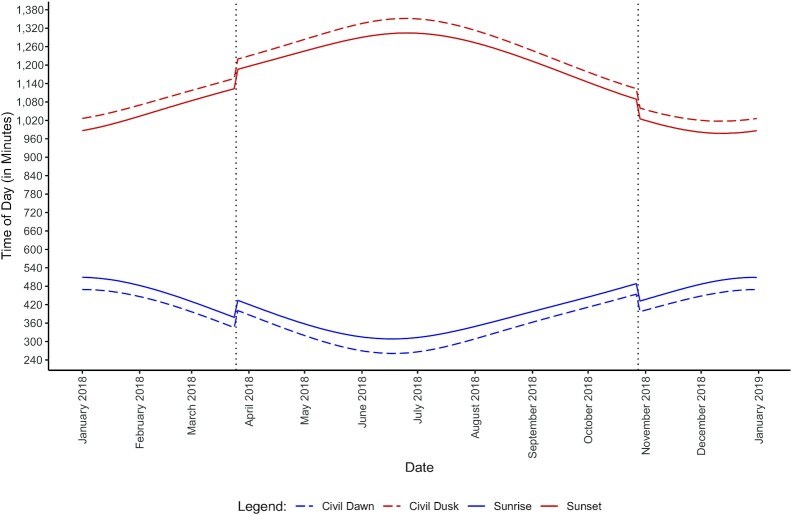
Average times of day for civil dawn, sunrise, sunset, and civil dusk in 2018.

In a first step, we contribute to the existing literature by providing more refined estimates for the impact of *daylight* (the time between sunrise and sunset) on bicycle ridership. In addition to previous studies, we also provide novel estimates for the impact of *twilight* (the time between civil dawn and sunrise as well as between sunset and civil dusk) on bicycle ridership. In order to identify these effects, we exploit 3 sources of exogenous variation in light conditions as part of our identification strategy (see also (8)). First, the timing of sunrise and sunset varies significantly over the course of the year, which leads to changes in light conditions for a given location and time of day (see Fig. 1). Second, the timing of sunrise and sunset varies between eastern and western regions, leading to changes in light conditions for a given day and time of day (see Figure S2A, Supplementary Material). Third, northern and southern regions differ from each other, as northern regions have relatively more daylight in summer months, and southern regions have relatively more daylight in winter months, again leading to changes in light conditions for a given day and time of day (see Figure S2B, Supplementary Material).

For the calculation of these effects, we use an extensive dataset of quarter-hourly bicycle counts from 146 automated counting stations in Germany. Using such finely structured bicycle-count data significantly improves the accuracy of estimating the time-sensitive effects of sunrise and sunset. In contrast to the literature, we moreover use hourly instead of daily weather control variables in order to improve accuracy. Our results are then calculated with negative binomial and log-linear regression models.

Exploiting the 3 exogenous types of variation in light conditions, we then find that daylight and twilight generally lead to higher bicycle ridership. The results depend on the type of bicycle traffic (utilitarian vs. mixed vs. recreational counting stations) and whether we consider the morning or the evening hours.

As a second step, we extend the literature by using our regression results to predict relative changes in overall bicycle ridership that would arise under an all-year DST or an all-year Standard Time (ST). The results show that, with respect to maximizing bicycle ridership, a time regime with an all-year DST would be better than a regime with an all-year ST and generate between 3.14 % and 3.37 % higher bicycle ridership. This is mainly due to increases in afternoon and evening ridership.

As a third step, we calculate how all-year time regimes would affect absolute bicycle ridership in Germany. We find that under an all-year DST, there would be 1.27–1.36 billion additionally cycled kilometers per year, compared to an all-year ST. This would lead to an increase in external benefits of 233.2–250.5 million Euros per year. As we do not consider private cost changes and our data do not enable accounting for modal shift effects, the overall economic effects are likely to be even greater.

The remainder of this paper is as follows. At first, we outline the data, as well as descriptive statistics for sun positions and light conditions. Then, we analyse the impact of daylight and twilight on bicycle ridership. Based on this, we predict relative changes in cycling that would arise under an all-year DST or an all-year ST. We then provide estimates for the changes in absolute bicycle ridership, and the external effects that would follow an implementation of all-year time regimes. The last section concludes.

## Data and Descriptive Statistics

### Bicycle counts, weather data, and calendar events

In order to measure the impact of clock changes on bicycle ridership, we use quarter-hourly bicycle counts from 146 automated bicycle counting stations in 23 German cities as our dependent variable.^1^ The geographic locations of these cities, as well as the number of bicycle counting stations per city, is displayed in Figure S1 (Supplementary Material). All bicycle counting stations of the sample were installed by EcoCounter and have a reported accuracy of above 95 %. Similar to Miranda-Moreno et al. (17), we scan the raw dataset for implausible extreme values and subsequently exclude them from the regression analysis. Such extreme values, as well as the missing values were evenly distributed across different counting stations, with no discernible pattern. Thus, the remaining data are comparable between counting stations and cities, as well as over time.

Our observation period spans the 2 years from 2017 January 1 to 2018 December 31. 124 of all counting stations report bicycle counts for at least 95 % of all 15-minute intervals that lie within the observation period. All in all, we have 9,241,099 quarter-hour observations of bicycle counts. Summary statistics for bicycle counts and other variables can be found in Table S1 (Supplementary Material).

To control for determinants of bicycle ridership, the bicycle count data are then merged with weather data from Germany’s National Meteorological Service (Deutscher Wetterdienst). This includes information on air temperature (in ^°^C), precipitation (in mm), wind speed (in m/s), and cloud coverage (in eights of the sky that are covered by clouds). For the regression models, we also include a quadratic term for the temperature, and we use 6 dummy variables for different levels of precipitation.^2^ Furthermore, the dummy variable *precipitation_lag* controls for rain during the last 3 hours. We also obtain data on general holidays, school holidays, and semester breaks from public sources, and include corresponding day-specific dummy variables in our dataset. Fixed effects for the stations, weekdays, hours, calendar weeks, and years of the sample are also included in our regression models.

Following the counter classification method outlined in Wessel (20), 96 of the 146 automated bicycle counting stations can be classified as measuring mainly utilitarian traffic, 25 as measuring mixed traffic, and 25 as measuring mainly recreational traffic.

### Sunrise and sunset data

The times of day for civil dawn, sunrise, sunset, and civil dusk, are calculated via the R package “suncalc” for each city of the sample. Civil dawn refers to the time of day when the ascending sun reaches an angle of 6° below the horizon. Now, the morning civil twilight begins and lasts until sunrise, which is the time of day when the upper rim of the sun appears on the horizon. In the evening, sunset is defined as the point when, for the descending sun, the upper rim of the sun is no longer visible on the horizon. This marks the start of the evening civil twilight, which lasts until the descending sun is at an angle of 6^°^ below the horizon. The times of day for these 4 events, depending on the specific day in 2018, and averaged over all cities of the sample, is displayed in Fig. 1. The solid lines refer to sunrise and sunset, whereas the dotted lines refer to civil dawn and civil dusk. The 2 vertical, dashed lines highlight the days when it is switched from ST to DST in spring, and from DST to ST in autumn. One can immediately see that the daylight length is greater in summer months, and lower in winter months. Although the daylight length is not affected by the implementation of DST, the distribution of daylight across different hours of the day is affected by this, which in the summer leads, c.p., to fewer daylight hours in the morning, but more in the evening. Regional differences for the times of sunrise and sunset are displayed in Figure S2 (Supplementary Material).

To control for the impact of daylight and twilight on bicycle ridership, we create the 2 variables *daylight* and *twilight*. The variable *daylight* reports the percentage of daylight for each 15-minute interval of the sample. This variable takes the value 0 if the upper rim of the sun is below the horizon for the whole 15 minutes, and takes the value 1 if the upper rim of the sun is visible for the whole 15 minutes. If the upper rim of the sun crosses the horizon within the 15-minute interval (either when ascending or when descending), the variable value is set as the share of minutes within the 15-minute interval for which the upper rim of the sun is visible.

**Fig. 2:**
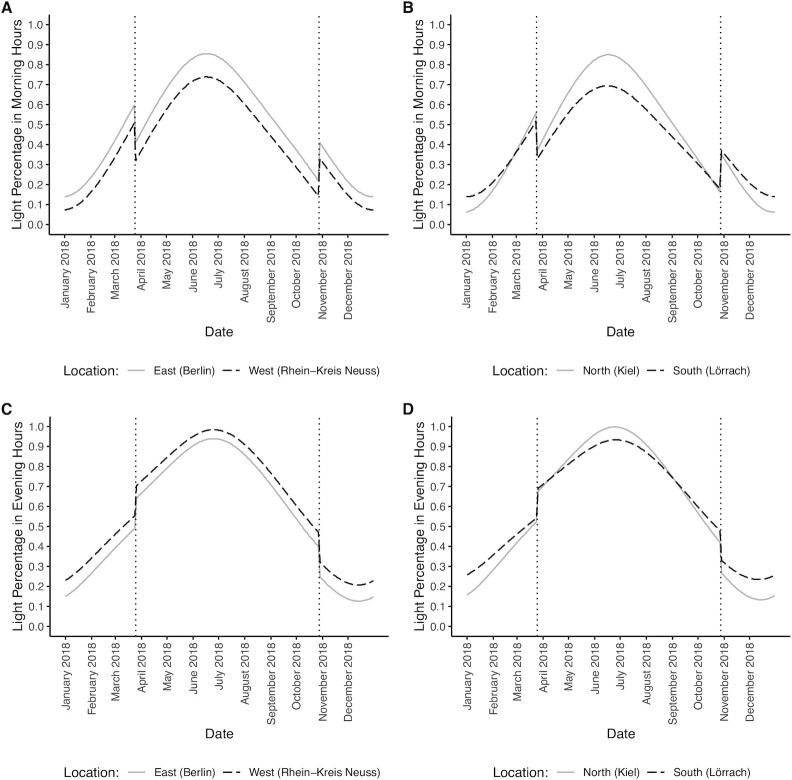
Daylight percentages for different locations in 2018. (**A**) Daylight percentage in morning hours (4:00–8:59) for the easternmost and the westernmost cities of the sample. (**B**) Daylight percentage in morning hours (4:00–8:59) for the northernmost and the southernmost cities of the sample. (**C**) Daylight percentage in evening hours (15:00–21:59) for the easternmost and the westernmost cities of the sample. (**D**) Daylight percentage in evening hours (15:00–21:59) for the northernmost and the southernmost cities of the sample.

The share of minutes with daylight is displayed in Fig. 2A and B for the morning hours from 4:00 to 8:59, and in Fig. 2C and D for the evening hours from 15:00 to 21:59. These figures show that the switch from ST to DST in the spring reduces the share of minutes with daylight in the morning hours, but in the evening hours, the share of minutes with daylight increases. The converse holds for the switch from DST to ST in autumn. Thus, DST impacts significantly on light conditions within morning and evening hours, and could thus have important ramifications for bicycle ridership.

Moreover, there are significant regional differences in the light percentage in morning and evening hours over the course of the year. In the morning hours, light percentage in the east is higher than in the west of Germany (Fig. 2A), and the opposite holds for evening hours (Fig. 2C). While these differences are fairly equal over the course of the year, the differences in light percentage between northern and southern regions depends on the time of year. In summer months, northern regions have a higher light percentage in both morning and evening hours than southern regions, but in winter months, it is the opposite (Fig. 2B and D).

In a similar vein to the *daylight*-variable, we create the variable *twilight*, which reports the share of minutes within each 15-minute interval that can be classified as twilight. This is the case if the upper rim of the sun is not above the horizon, and the sun position is not below 6° under the horizon. We then exploit the variations in daylight and twilight that occur over the course of the year, as well as those caused by differences in the longitude and latitude of the bicycle counters, in order to estimate the impact of these 2 variables on bicycle ridership.

Moreover, we account for the fact that there may be interdependencies between cycling in the morning and cycling in the evening for commuting trips (e.g. commuting to work, school, or university). People who commute to work by car or bus usually cannot use their bicycle for their return trip. Thus, less daylight in the morning could lead not only to less cycling in the morning, but thereby also to less cycling in the evening. Hence, we control for (i) the impact that daylight during the morning rush hours (7:00–8:59, based on actual traffic counts) has on cycling in the evening, and (ii) the impact that daylight during the evening rush hours (16:00–18:59) has on cycling in the morning. To this end, the variable *morning_rush_daylight* reflects the share of minutes with daylight during the morning rush hours on a given day and in a given city. The variable *evening_rush_daylight* is then created in a similar way for the evening rush hours.

## Estimation of the Impact of Daylight and Twilight on Bicycle Ridership

### Basic regression model

In the following analysis, we estimate the impact of *daylight* and *twilight* on bicycle ridership. Hereby, the regression coefficient of *daylight* indicates the percentage change in bicycle ridership if the upper rim of the sun is visible for an entire quarter-hour interval, compared to when the sun is more than 6^°^ below the horizon for an entire quarter-hour interval (i.e. our reference category). The regression coefficient of *twilight* indicates the percentage change in bicycle ridership if the upper rim of the sun is below the horizon, but the sun is less than 6° below the horizon for an entire quarter-hour interval, compared to when the sun is more than 6^°^ below the horizon for an entire quarter-hour interval.

As is common practice in the literature on determinants of bicycle ridership, we use negative binomial and log-linear regression models in order to estimate the impact of daylight on bicycle ridership. Both models are regularly used for bicycle count data and have been shown to provide a good fit for the data (21). For reasons of clarity and comprehensibility, we focus on the negative binomial regression model within this subsection, and briefly discuss the log-linear regression model, as well as a more differentiated version of the negative binomial regression model as part of the sensitivity analyses in the next subsection.

We run separate negative binomial regression models for morning hours (2:00–9:59) and for evening hours (15:00-22:59).^3^ Moreover, we differentiate between mainly utilitarian, mixed, and mainly recreational bicycle counting stations, as these types of traffic have generally been shown to behave differently (17).^4^ Against this backdrop, the 2 variables that account for links between morning and evening cycling (*morning_rush_daylight* and *evening_rush_daylight*) are used in the analyses of bicycle ridership at utilitarian counting stations, i.e. where commuting trips are very common. At mixed and recreational counting stations, however, commuting trips are of rather minor importance, thus not warranting the inclusion of these 2 variables in the respective regression models.^5^

The results of the negative binomial regression models for these 6 subsets are then reported in Table 1. We find that daylight increases utilitarian traffic in morning hours by 26.7 % (Column 1) and mixed traffic by 19 % (Column 2), but does not impact on recreational traffic (Column 3). Utilitarian and mixed bicycle traffic in morning hours is also positively affected by twilight, but to a lesser degree than by daylight. Again, recreational bike traffic in morning hours appears not to be significantly affected by twilight. These results could be explained by the usual timing of utilitarian and recreational trips. Whereas utilitarian bicycle trips are common in morning hours (e.g. commuting to work) and could more easily be influenced by the presence of daylight or twilight (e.g. substitution by another means of transport), recreational trips are quite uncommon in morning hours, irrespective of the presence of daylight or twilight.

**Table 1.** Basic regression per counter type (negative binomial).

Dependent variable:	Counts					
	Morning hours (2:00–9:59)			Evening hours (15:00-22:59)		
	Utilitarian	Mixed	Recreational	Utilitarian	Mixed	Recreational
	(1)	(2)	(3)	(4)	(5)	(6)
Daylight	0.2671***	0.1901***	0.0733	0.2124***	0.3393***	0.9739***
	(0.0205)	(0.0394)	(0.0614)	(0.0158)	(0.0690)	(0.0686)
Twilight	0.1782***	0.1320***	0.0770	0.1881***	0.2600***	0.6647***
	(0.0127)	(0.0285)	(0.0516)	(0.0132)	(0.0568)	(0.0500)
	0.5129**			−0.2531		
	(0.2156)			(0.2202)		
Temperature	0.0263***	0.0252***	0.0357***	0.0510***	0.0530***	0.1518***
	(0.0025)	(0.0022)	(0.0048)	(0.0026)	(0.0066)	(0.0093)
Temperature^2^	−0.0002**	7.11 × 10^−5^	0.0003*	−0.0007***	−0.0005***	−0.0027***
	(7.97 × 10^−5^)	(0.0001)	(0.0002)	(7.24 × 10^−5^)	(0.0002)	(0.0003)
Light_drizzle	−0.1627***	−0.1627***	−0.2871***	−0.1780***	−0.2104***	−0.4010***
	(0.0074)	(0.0168)	(0.0170)	(0.0066)	(0.0281)	(0.0177)
Strong_drizzle	−0.2105***	−0.2375***	−0.3220***	−0.1932***	−0.2192***	−0.3911***
	(0.0121)	(0.0253)	(0.0329)	(0.0071)	(0.0254)	(0.0419)
Light_rain	−0.2951***	−0.3230***	−0.3680***	−0.2188***	−0.3036***	−0.4947***
	(0.0146)	(0.0376)	(0.0345)	(0.0090)	(0.0312)	(0.0322)
Moderate_rain	−0.4075***	−0.3762***	−0.4565***	−0.2356***	−0.2859***	−0.3644***
	(0.0178)	(0.0533)	(0.0668)	(0.0095)	(0.0267)	(0.0421)
Strong_rain	−0.5316***	−0.5387***	−0.7746***	−0.1857***	−0.1249**	−0.2149***
	(0.0370)	(0.0727)	(0.1210)	(0.0277)	(0.0627)	(0.0584)
Heavy_rain	−0.4555**	−0.8882***	0.0863	−0.1338***	−0.1105*	0.0261
	(0.2026)	(0.1575)	(0.8488)	(0.0261)	(0.0646)	(0.1221)
Precipitation_lag	−0.2289***	−0.2666***	−0.5031***	−0.3114***	−0.3567***	−0.6934***
	(0.0108)	(0.0134)	(0.0336)	(0.0089)	(0.0289)	(0.0198)
Cloudiness	−0.0124***	−0.0140***	−0.0285***	−0.0186***	−0.0222***	−0.0530***
	(0.0009)	(0.0016)	(0.0022)	(0.0011)	(0.0028)	(0.0030)
Windspeed	−0.0197***	−0.0227***	−0.0269***	−0.0267***	−0.0293***	−0.0573***
	(0.0015)	(0.0040)	(0.0027)	(0.0012)	(0.0044)	(0.0055)
General_holidays	−0.8422***	−0.9513***	−0.5988***	−0.5634***	−0.3600***	0.3513***
	(0.0549)	(0.0832)	(0.0793)	(0.0245)	(0.0619)	(0.0461)
School_holidays	−0.2203***	−0.1728***	−0.2048***	−0.1774***	−0.1065***	−0.0172
	(0.0075)	(0.0103)	(0.0224)	(0.0079)	(0.0061)	(0.0258)
Semester_break	−0.0224**	−0.0063	0.0519	−0.0411***	−0.0541**	0.1006**
	(0.0088)	(0.0224)	(0.0444)	(0.0090)	(0.0249)	(0.0513)
Station FE	Yes	Yes	Yes	Yes	Yes	Yes
Weekday FE	Yes	Yes	Yes	Yes	Yes	Yes
Hour FE	Yes	Yes	Yes	Yes	Yes	Yes
Week FE	Yes	Yes	Yes	Yes	Yes	Yes
Year FE	Yes	Yes	Yes	Yes	Yes	Yes
Observations	2,017,080	465,204	556,247	2,016,511	465,545	556,813
Squared correlation	0.71511	0.73053	0.60069	0.81142	0.70368	0.74932

Standard errors are clustered by counting station.

Significance codes: ***: 0.01, **: 0.05, and *: 0.1.

For utilitarian bicycle traffic in evening hours (Column 4), the positive impact of daylight is slightly less pronounced than in the morning hours, but the impact of twilight on utilitarian bicycle ridership is fairly similar between morning and in evening hours. For mixed and recreational bicycle traffic (Columns 5 and 6), however, the impact of daylight and twilight is much stronger for evening than for morning hours. Such trips can often be rescheduled or cancelled easily, so that they are more dependent on the overall conditions for cycling, which are generally better during daylight or twilight than in darkness.

We also find that daylight in evening rush hours has a statistically significant and positive effect on utilitarian bicycle ridership in the morning (Column 1). This indicates that people who can expect to get home from work in daylight are more likely to choose their bicycle for their trip to work. We find no significant effect of daylight in morning rush hours on utilitarian bicycle ridership in the evening.

### Sensitivity analyses

In order to verify the aforementioned results, we conduct 2 types of sensitivity analyses. First, we use a log-linear regression to test if our results might be driven by the used regression method. The detailed results of the log-linear regression models can then be found in Table S2 (Supplementary Material). Recreational bicycle traffic in the morning hours is now positively affected by daylight, but there is a slightly negative impact of twilight. Also, the positive impact of twilight on recreational bicycle traffic in evening hours is no longer significant. Otherwise, the results are mostly comparable to the negative binomial regression models and thus confirm our earlier findings.

Second, we test if the impact of daylight and twilight varies over different seasons, and if the strength of the impact is additionally affected by the presence of rain or of a clouded sky. For this, we split the original *daylight*-variable into 4 season-specific variables. The variable *daylight_spring*, for example, then takes the value of the original *daylight*-variable on days that lie in spring, and 0 otherwise. Moreover, the 2 interaction terms *daylight_rainy* and *daylight_clouded* estimate the changes in the strength of the impact of daylight if it is rainy (*precipitation* > 0) or if more than half of the sky is covered by clouds (*cloudiness* ≥ 5). Similar transformations are done for the *twilight*-variable. The detailed regression results can then be found in Table S3 (Supplementary Material).

The results show that the impact of light conditions on bicycle riderships appears to vary over the 4 seasons. Utilitarian ridership is generally more positively affected by daylight and twilight on autumn or winter days, compared to spring and summer days. Similarly, the positive impact of daylight on mixed traffic is least pronounced during the summer. In contrast to this, recreational traffic in morning hours is only significantly affected by daylight in spring and summer, but not in autumn or winter. Recreational traffic in evening hours is, however, strongly affected by daylight in all 4 seasons.

The impact of twilight on mixed and recreational morning traffic is generally more pronounced during autumn and winter days. Mixed and recreational evening traffic is more increased by twilight in summer and autumn days than in winter and spring.

Additionally, we can see that rain generally decreases the positive impact of daylight and twilight, and a clouded sky lowers the positive impact of twilight on cycling levels in the evening.

## Prediction of Bicycle Ridership Under All-Year ST and All-Year DST

Now that we have calculated the impact of daylight and twilight on bicycle ridership, we can analyze how bicycle ridership in 2017 and 2018 would have unfolded under an all-year ST or under an all-year DST. The results can help to evaluate whether either an all-year ST, or an all-year DST would lead to higher bicycle ridership if governments decided to abolish the bi-annual clock change. Accordingly, we predict the number of bicycle counts per 15-minute interval for 3 different cases: (i) the current time regime with bi-annual clock change, (ii) an all-year implementation of DST, and (iii) an all-year implementation of ST.

Using the regression coefficients outlined in Table 1 and Tables S2 and S3 (Supplementary Material), we predict bicycle ridership for these 3 cases by manipulating the times of civil dawn, sunrise, sunset, and civil dusk in each of the 6 subsets accordingly. All other variables are not changed, implying that they take the actual values that were observed in 2017 and 2018. Moreover, we run the same regression models that are used in Table 1 and Tables S2 and S3 (Supplementary Material) for all remaining data and predict bicycle ridership for these observations in a similar manner.^6^

The prediction results for the 3 different regression models can be found in Table 2. Over the whole year, an all-year DST would increase overall bicycle ridership by 2.00 %–2.12 % compared to the current regime, and an all-year ST would decrease bicycle ridership by 1.10 %–1.27 %. The results thus underline that if the current bi-annual clock change were abolished and 1 permanent time regime were to be implemented, an all-year DST would be more beneficial for fostering bicycle ridership. Under an all-year DST, bicycle ridership would be 3.14 %–3.37 % higher than under an all-year ST.

**Table 2.** Overview of prediction results.

	DST vs. current regime (%)			ST vs. current regime (%)			DST vs. ST (%)		
	NB-Base	Log-Lin	NB-Diff	NB-Base	Log-Lin	NB-Diff	NB-Base	Log-Lin	NB-Diff
All months	2.12	2.01	2.00	−1.21	−1.27	−1.10	3.37	3.32	3.14
Utilitarian	2.37	2.23	2.23	−1.23	−1.32	−1.12	3.65	3.60	3.39
Mixed	0.39	0.43	0.43	−0.82	−0.91	−0.75	1.22	1.35	1.18
Recreational	0.71	0.54	0.78	−1.86	−1.14	−1.75	2.62	1.69	2.57
Morning hours	2.41	2.14	1.93	−1.00	−0.47	−0.87	3.44	2.62	2.82
Evening hours	3.45	3.14	3.43	−2.15	−2.28	−1.98	5.73	5.56	5.52

NB-Base refers to predictions based on the basic negative binomial regression model. Log-Lin refers to the log-linear regression model, and NB-Diff refers to the more differentiated negative binomial regression model outlined in the sensitivity analysis.

**Fig. 3:**
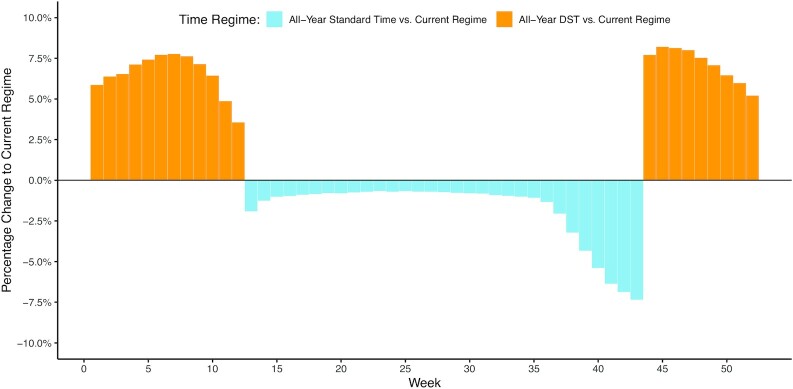
Weekly predictions for basic negative binomial regression model (in %).

Figure 3 illustrates the weekly percentage changes in bicycle ridership when switching from the current regime with a bi-annual clock change to a regime with an all-year DST (in orange), as well as when switching from the current regime to a regime with an all-year ST (in skyblue). We can see that for an all-year DST, bicycle ridership would increase in winter months, and in the summer months there would be no change, as DST is currently already implemented for those months. For an all-year ST, bicycle ridership would decrease in summer months, and there would be no change in the winter months as ST is already implemented for those months. We can also see that the differences between the current and the all-year time regimes are generally most pronounced shortly before the autumn clock change.

**Fig. 4:**
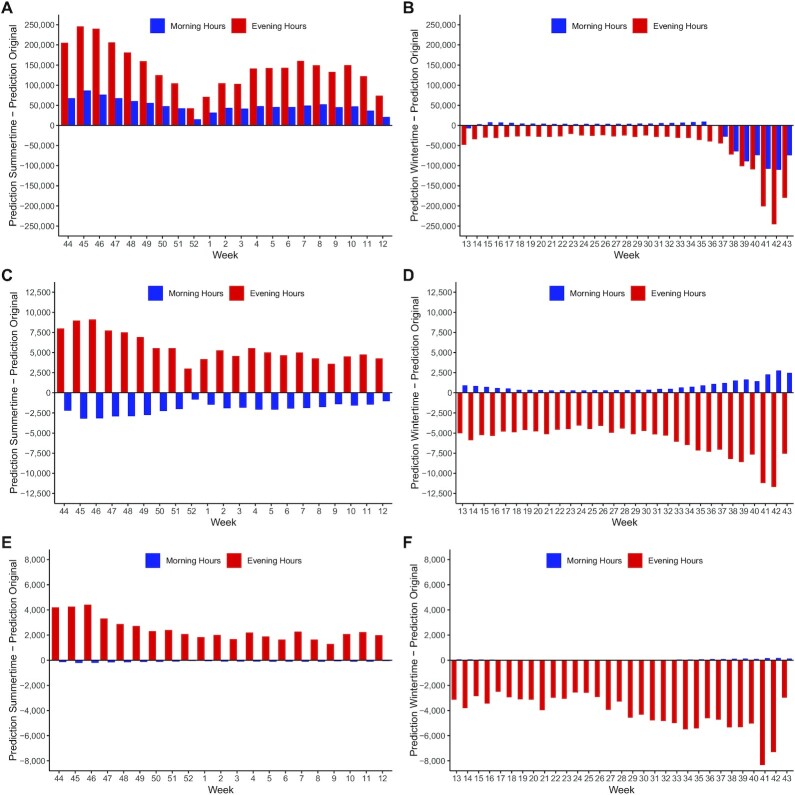
Weekly predictions for different counter types and daytimes (in absolute values, based on the negative binomial regression model). (**A**) Changes in weekly utilitarian bicycle traffic with an all-year DST in winter months. (**B**) Changes in weekly utilitarian bicycle traffic with an all-year ST in summer months. (**C**) Changes in weekly mixed bicycle traffic with an all-year DST in winter months. (**D**) Changes in weekly mixed bicycle traffic with an all-year ST in summer months. (**E**) Changes in weekly recreational bicycle traffic with an all-year DST in winter months. (**F**) Changes in weekly recreational bicycle traffic with an all-year ST in summer months.

To shed more light on the mechanisms through which different time zones impact on bicycle ridership, we turn to Fig. 4, in which absolute gains and losses of different time regime implementations are separated over the weeks under ST and the weeks under DST, as well as over the 3 different counter types. These absolute changes for morning hours (blue) and evening hours (red) are based on the negative binomial regression model, but the main insights also remain valid for the predictions based on the log-linear and the more differentiated negative binomial regression model. In Fig. 4A, we can see that an implementation of DST in winter months would lead to more utilitarian bicycle traffic in both morning and evening hours. Here, a later sunset would lead to more evening time with daylight, and thus to more utilitarian bicycle traffic in evening hours. Apparently, the positive impact of being able to get home from work in daylight outweighs the negative impact of less daylight in morning hours, thereby leading to a net increase in morning cycling. Figure 4B then illustrates the impact of a permanent implementation of ST on utilitarian bicycle ridership in summer months, separated for morning and evening hours. Due to the earlier sunset, there is less daylight in evening hours, consequently reducing bicycle ridership in the evening hours. Less daylight in evening hours also implies that people cannot always get home from work in daylight, and, anticipating this, they are also less likely to ride their bikes to work in the morning. The negative impact on both morning and evening cycling is especially pronounced shortly before the autumn clock change.

For mixed bicycle traffic (Fig. 4C and D) and for recreational bicycle traffic (Fig. 4E and F), the absolute impact on evening hours is stronger than the impact on morning hours, resulting in positive net effects when switching to DST in winter months, and in negative net effects when switching to ST in summer months. The net effects are generally more pronounced for recreational bicycle traffic than for mixed bicycle traffic.

## Impact on German Cycling Levels, Economic Effects, and Discussion

### Effects on overall German cycling levels

In the previous section, we have seen that an all-year implementation of DST would lead to higher cycling levels than the current time regime or an all-year implementation of ST. Although the expected percentage changes are relatively small at first glance, the implementation of different time regimes would still translate to significant absolute changes. To illustrate this, we combine the estimated percentage changes in bicycle ridership that were outlined in Table 2 with data on overall mobility from Germany in 2017. The data on overall mobility is based on the nationwide household mobility survey “Mobilität in Deutschland” (Engl.: Mobility in Germany), which is commissioned by the Federal Ministry of Transport and Digital Infrastructure (22).

In 2017, the German people made 28 million bike trips with a total length of 112 million bicycle kilometers per day. Assuming that the change in counted bicycles, which is based on data from 146 counting stations in 23 German cities, is representative of overall German bicycle traffic, and that cycled kilometers increase in the same manner as the number of counted bicycles, we can estimate changes in overall bicycle kilometers that would accrue if all-year time regimes were to be implemented. The absolute changes, which are displayed in the upper part of Table 3, are indeed not negligible and amount to an increase of roughly 1.27–1.36 billion cycled kilometers per year if we compare an all-year DST to an all-year ST.

**Table 3.** Absolute changes in bicycle ridership and external effects.

	Prediction method		
	NB-Base	Log-Lin	NB-Diff
*1) Change in absolute kilometers (in pkm p.a.)*			
DST vs. current system	866,656,000	821,688,000	817,600,000
ST vs.c urrent system	−494,648,000	−519,176,000	−449,680,000
DST vs. ST	1,361,304,000	1,340,864,000	1,267,280,000
*2) External effects (in € p.a.)*			
DST vs. current system	159,464,704 €	151,190,592 €	150,438,400 €
ST vs. current system	−91,015,232 €	−95,528,384 €	−82,741,120 €
DST vs. ST	250,479,936 €	246,718,976 €	233,179,520 €

NB-Base refers to predictions based on the basic negative binomial regression model. Log-Lin refers to the log-linear regression model, and NB-Diff refers to the more differentiated negative binomial regression model outlined in the sensitivity analysis.

Based on German mobility data in 2017 from Nobis (22), external cost factors from Gössling (23), and own calculations.

As outlined in the text, the presented external effects should be viewed as lower-bound estimates. Moreover, the overall economic effects—including the impact on private costs—are likely to be higher, but cannot be calculated due to insufficient data for other transport modes.

### Approximation of economic effects

Our next step is to provide estimates for the economic consequences of these changes in cycled kilometers. To this end, we only focus on the external costs and benefits of cycling itself. We intentionally disregard the impact on private costs for 2 reasons. First, newly generated *utilitarian* bicycle traffic (e.g. commuting) would most likely not be induced traffic, but due to a modal shift from other transport modes. As private costs for cycling are relatively low compared to other transport modes (23), our approach ensures that we do not overvalue the overall economic effects. Second, Gössling (23) show that by far the largest private cost component of cycling is travel time cost, but, for *recreational* traffic, it would be hard to argue that cyclists perceive their travel time as a cost rather than a benefit, thus casting doubt on the appropriateness of this cost component for recreational bicycle traffic. In conclusion, not considering private costs ensures that our monetary estimates of external effects could be regarded as lower-bound estimates for the overall economic effects of different time regimes on cycling.

When it comes to analyzing the external effects, we suppose that all changes in cycled kilometers are because of induced bicycle traffic, and not because of modal shifts. This is of course rather unrealistic, but we have no reliable estimates for modal shifts caused by changes in daylight and twilight conditions. Considering that the external effects of transport modes such as road traffic or public transport are more negative than for cycling (23,24), our estimates for the external effects of time-regime changes on cycling could, therefore, be regarded as lower-bound estimates.

To monetize the changes in cycled kilometers that are outlined in the upper part of Table 3, we follow Gössling (23), who estimated the overall private and external costs for each passenger kilometer when traveling by car, bicycle, or when walking. For cycling, external costs are lower than external benefits, resulting in a net external benefit of 0.184 Euro for each passenger kilometer by bike. This positive effect is mainly due to health effects (e.g. lower costs for medical treatments).

Multiplying this factor by the annual changes in bicycle passenger kilometers, we obtain the annual external effects that are displayed in the lower part of Table 3. Introducing an all-year DST instead of an all-year ST could then lead to an increase in external benefits of around 233.2–250.5 million Euros per year. These results underline that the choice of an all-year time regime can have important economic consequences, even when only looking at the transport mode of cycling. As outlined above, the external benefits might in fact be even higher if the effects of modal shifts were included in the analysis. Moreover, when including private costs in the analysis as well, the overall economic effects should be higher than the presented external effects. It is also important to note that the external benefits in Table 3 would accrue each year, thereby further increasing the relevance of choosing the *best* time regime.

### Discussion

With respect to cycling, we can conclude that an all-year implementation of DST is superior to the current time regime and to an all-year implementation of ST. This is reflected in higher cycling levels and higher external benefits.

Due to the focus on cycling and insufficient data for other transport modes, however, we cannot estimate modal shifts that would follow an abolishment of the bi-annual clock change. This implies that we cannot calculate economic effects for the whole transport sector, and that our estimates would have to be viewed as lower-bound estimates for external effects rather than the actual overall effects. Thus, modeling the impact of all-year time regimes on other transport modes, and subsequently modal shift effects, would be an interesting area for further research and allow monetizing the effects for the whole transport sector.

Besides not accounting for potential modal shifts in our economic appraisal, another limitation of our analysis is that changes in the daily distribution of daylight could lead to changes in traffic crash rates, and subsequently on social costs. It should, however, be noted that Coate and Markowitz (25) or Bünnings and Schiele (8) find that an all-year implementation of DST would lead to lower crash rates than an all-year ST, thereby supporting the direction of our results even further.

Another interesting aspect that could be affected by the choice of time regime would be congestion during rush hours. Especially in winter months, sunrise and sunset lie within the morning and evening rush hours, so that the choice of the time regime could change the light conditions and subsequently impact on bicycle ridership within these hours. As outlined in Fig. 4A for utilitarian traffic in winter months, an all-year DST would generally lead to an increase in cycling in both morning hours and evening hours. Our results thus suggest that, if the change in bicycle ridership is at least partially due to modal shifts from private automobile transport to cycling, an all-year DST might reduce congestion in morning and evening rush hours. Further research in this area could, therefore, provide helpful insights for tackling congestion.

The impact on cycling levels and the changes in external effects that were estimated in this paper refer to Germany alone. As outlined in the Introduction, however, an end to the bi-annual clock change was planned to be an EU-wide project and the choice of an all-year time regime would subsequently have ramifications for the other countries of the EU as well. Depending on their geographical location and bicycle-affinity, the estimated effects could be more or less pronounced than for Germany, but the overall direction of results should be comparable. Thus, the EU-wide effects of choosing an all-year time regime should be significantly higher than the effects reported herein for Germany.

## Conclusions

The promotion of cycling is an important cornerstone for a more sustainable future (14). In this paper, we first show in which ways daylight and twilight can impact on bicycle ridership. We find that better light conditions generally lead to higher levels of cycling. The extent of this effect depends on the type of traffic (e.g. utilitarian vs. recreational) and whether we look at morning or at evening hours.

Second, we analyze how the discussed abolishment of the bi-annual clock change in the EU would impact on cycling levels. Therefore, we use the estimated effects of daylight and twilight on bicycle ridership, as well as datasets with adjusted sunrise and sunset times. An all-year implementation of DST would then lead to an increase in cycling levels of around 3.14 %–3.37 %, compared to an all-year ST.

Third, we calculate the absolute changes in German bicycle ridership and find that an all-year implementation would lead to an increase of around 1.27–1.36 billion cycled kilometers per year, compared to an all-year ST. This would translate into external benefits of around 233.2–250.5 million Euros per year in Germany. As we do not consider private cost changes and our data do not enable accounting for modal shift effects, the overall economic effects are likely to be even higher.

We can thus conclude that an all-year implementation of DST is superior to the current time regime and to an all-year implementation of ST. This is reflected in higher cycling levels and higher external benefits. Of course, the choice for a time regime should not be based on cycling levels alone, but when it comes to promoting cycling, DST would be the best choice.

## Supplementary Material

pgab006_Supplemental_File

## Data Availability

The data of our research project cannot be shared. The bicycle count data is proprietary and owned by the respective cities. Thus, we are not the legal owner of the data and consequently not authorized to publish the data to the broader public. However, all bicycle count data can be obtained directly from the respective cities. For this purpose, we have contacted the cities via the contact addresses provided on their websites.

## Footnotes

1 The raw data, i.e. the number of bicycles per quarter hour, are property of the respective cities, but were kindly provided by them.

2 Our scale is adapted from the rain intensity scale presented by Tokay and Short (18), which is representative, according to Dunkerley (19). The actual boundaries of our classification are then based on Wessel (20): light drizzle (precipitation— $\lt \, 0.5\, \rm {mm/h}$), strong drizzle ($0.5\, \rm{mm/h}\, \leq$ precipitation $\lt \, 1\, \rm{mm/h}$), light rain ($1\, \rm {mm/h} \, \leq$ precipitation $\lt \, 2\, \rm{mm/h}$), moderate rain ($2\, \rm{mm/h} \, \leq$ precipitation $\lt \, 5\, \rm{mm/h}$), heavy rain ($5\, \rm{mm/h} \, \leq$ precipitation $\lt \, 10\, \rm{mm/h}$), and very heavy rain ($10\, \rm{ mm/h} \, \leq$ precipitation).

3 These timeframes are chosen in such a way that they encompass the earliest possible time of the civil dawn under an all-year ST (2:44) and the latest possible time of the sunrise under an all-year DST (9:41) for the morning hours, as well as the earliest possible time of sunset under an all-year ST (15:49) and the latest possible time of civil dusk under an all-year DST (22:55) for evening hours.

4 For the counter classification, we follow the method outlined in Wessel (20). We choose to run separate regressions for each counter type to better capture the idiosyncrasies of each type of traffic (e.g. different reactions to bad weather or public holidays). A regression model that combines all types of traffic would necessitate an abundance of dummy variables and interaction terms, thereby reducing model clarity and increasing the risk of overfitting the regression model.

5 To verify this, we also tested the regression models for mixed counting stations with these 2 variables, but found no statistically significant effect of either variable. To reduce the risk of overfitting the models, we subsequently exclude these 2 variables from the regression models for mixed counting stations. For recreational counting stations, there would be even less justification for including these 2 variables, as recreational counting stations are defined to have even less commuting traffic than mixed counting stations.

6 The remaining data consist of observations in nonmorning and nonevening hours that are either always darker than civil dawn/civil dusk, or always with daylight. Subsequently, the variables *daylight* and *twilight* cannot be used in this regression analysis, because of perfect multicollinearity. The results of this regression analyses are available upon request.

## References

[1] European Commission . 2018. Summertime Consultation: 84% want Europe to stop changing the clock.

[2] Martín-Olalla JM . 2019. The long term impact of daylight saving time regulations in daily life at several circles of latitude. Sci Rep. 9, 18466. 31804602 10.1038/s41598-019-54990-6 PMC6895179

[3] Roenneberg T , Winnebeck EC, Klerman EB. 2019. Daylight saving time and artificial time zones – a battle between biological and social times. Front Physiol. 10, 944. 31447685 10.3389/fphys.2019.00944 PMC6692659

[4] Kotchen MJ , Grant LE. 2011. Does daylight saving time save energy? Evidence from a natural experiment in Indiana. Rev Econ Stat. 93, 1172–1185.

[5] Doleac JL , Sanders NJ. 2015. Under the cover of darkness: how ambient light influences criminal activity. Rev Econ Stat. 97, 1093–1103.

[6] Smith AC . 2016. Spring forward at your own risk: daylight saving time and fatal vehicle crashes. Am Econ J Appl Econ. 8, 65–91.

[7] Ferguson SA , Preusser DF, Lund AK, Zador PL, Ulmer RG. 1995. Daylight saving time and motor vehicle crashes: the reduction in pedestrian and vehicle occupant fatalities. Am J Pub Health. 85, 92–95. 7832269 10.2105/ajph.85.1.92 PMC1615292

[8] Bünnings C , Schiele V. 2021. Spring forward, don’t fall back: the effect of daylight saving time on road safety. Rev Econ Stat. 103, 165–176.

[9] Uttley J , Fotios S. 2017. Using the daylight savings clock change to show ambient light conditions significantly influence active travel. J Environ Psychol. 53, 1–10.

[10] Fotios S , Qasem H, Cheal C, Uttley J. 2017. A pilot study of road lighting, cycle lighting and obstacle detection. Light Res Technol. 49, 586–602.

[11] Boyce P , Eklund N, Hamilton B, Bruno L. 2000. Perceptions of safety at night in different lighting conditions. Int J Light Res Technol. 32, 79–91.

[12] Johansson Ö , Wanvik PO, Elvik R. 2009. A new method for assessing the risk of accident associated with darkness. Accident Anal Prevent. 41, 809–815. 10.1016/j.aap.2009.04.003 19540970

[13] Pucher J , Buehler R. 2017. Cycling towards a more sustainable transport future. Transp Rev. 37, 689–694.

[14] Brand C , et al. 2021. The climate change mitigation effects of daily active travel in cities. Transp Res Part D Transp Environ. 93, 102764.

[15] Fotios S , Uttley J, Fox S. 2017. A whole-year approach showing that ambient light level influences walking and cycling. Light Res Technol. 51, 55–64.

[16] Goodman A , Page AS, Cooper AR. 2014. Daylight saving time as a potential public health intervention: an observational study of evening daylight and objectively-measured physical activity among 23,000 children from 9 countries. Int J Behav Nutr Phys Act. 11, 84. 25341643 10.1186/1479-5868-11-84 PMC4364628

[17] Miranda-Moreno LF , Nosal T, Schneider RJ, Proulx F. 2013. Classification of bicycle traffic patterns in five North American cities. Transp Res Rec. 2339, 68–79.

[18] Tokay A , Short DA. 1996. Evidence from tropical raindrop spectra of the origin of rain from stratiform versus convective clouds. J Appl Meteorol. 35, 355–371.

[19] Dunkerley D . 2008. Rain event properties in nature and in rainfall simulation experiments: a comparative review with recommendations for increasingly systematic study and reporting. Hydrol Process. 22, 4415–4435.

[20] Wessel J . 2020. Using weather forecasts to forecast whether bikes are used. Transp Res Part A Pol Pract. 138, 537–559.

[21] Nordback KL . 2012. Estimating annual average daily bicyclists and analyzing cyclist safety at urban intersections. [PhD thesis]. [Denver (CO)]: University of Colorado Denver.

[22] Nobis C . 2019. Mobilität in Deutschland – MiD Analysen zum Radverkehr und Fußverkehr. Studie von infas, DLR, IVT, und infas 360 im Auftrag des Bundesministeriums für Verkehr und digitale Infrastruktur (FE-Nr. 70.904/15). Technical report, Bonn, Berlin: Bundesministeriums für Verkehr und digitale Infrastruktur.

[23] Gössling S , Choi A, Dekker K, Metzler D. 2019. The social cost of automobility, cycling and walking in the European Union. Ecol Econ. 158, 65–74.

[24] van Essen H , et al. 2019. Handbook on the external costs of transport (Version 2019). Brussels, European Union.

[25] Coate D , Markowitz S. 2004. The effects of daylight and daylight saving time on US pedestrian fatalities and motor vehicle occupant fatalities. Accid Anal Prevent. 36, 351–357. 10.1016/S0001-4575(03)00015-0 15003579

