# Supplementary material for: Cycling in the dark – the impact of Standard Time and Daylight Saving Time on bicycle ridership
Source: PNAS Nexus. 2022 Mar 2;1(1):pgab006. doi: 10.1093/pnasnexus/pgab006 (PMC9802456; doi:10.1093/pnasnexus/pgab006)
Supplement: pgab006_Supplemental_File [file pgab006_supplemental_file.pdf]

Supplemental Material

Table S1: Summary Statistics

Variable	N	Mean	St. Dev.	Min	Q25	Median	Q75	Max
cloudiness	459,686	5.62	3.02	0.00	3.00	7.00	8.00	8.00
counts	9,241,099	20.60	33.80	0	1	7	26	974
daylight	465,344	0.46	0.49	0	0	0	1	1
evening_peak_daylight	15,564	0.74	0.34	0.00	0.39	1.00	1.00	1.00
general_holidays	15,564	0.03	0.17	0	0	0	0	1
morning_peak_daylight	15,564	0.80	0.26	0.16	0.59	1.00	1.00	1.00
precipitation	463,321	0.07	0.51	0.00	0.00	0.00	0.00	52.90
precipitation_lag	463,132	0.06	0.24	0.00	0.00	0.00	0.00	1.00
school_holidays	15,564	0.24	0.43	0	0	0	0	1
semester_break	15,564	0.32	0.47	0	0	0	1	1
temperature	463,803	11.03	7.98	−18.20	5.00	10.90	16.80	37.30
twilight	465,344	0.14	0.31	0	0	0	0	1
windspeed	463,501	3.21	1.96	0.00	1.80	2.80	4.20	22.30

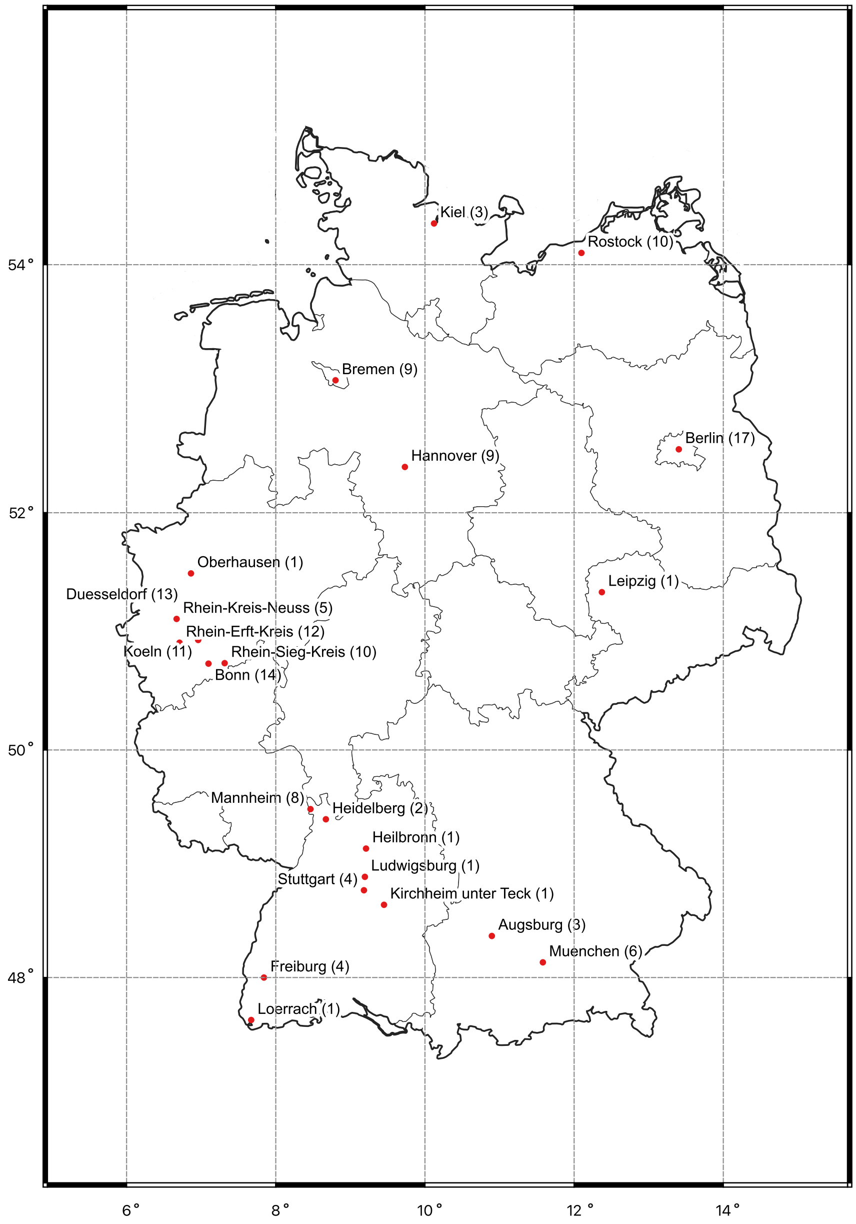

Figure S1: Cities and Counting Stations

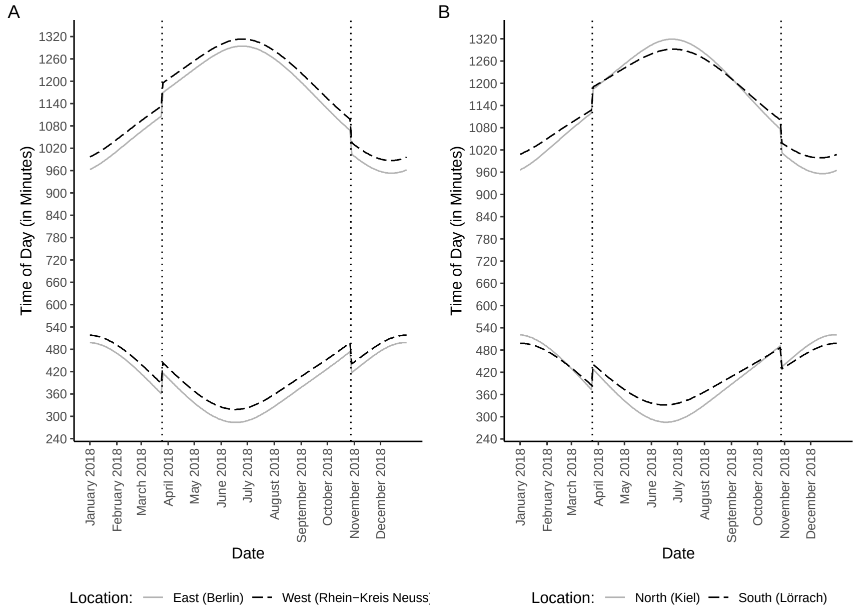

Figure S2: Sunrise and Sunset for Different Locations. (*A*) Sunrise and sunset times for the easternmost and westernmost cities of the sample. (*B*) Sunrise and sunset times for the northernmost and southernmost cities of the sample.

Table S2: Sensitivity Analysis (Log-Linear Regression Model)

Dependent Variable:	log(counts_adj)					
	Morning Hours (2:00–9:59)			Evening Hours (15:00-22:59)		
	Utilitarian (7)	Mixed (8)	Recreational (9)	Utilitarian (10)	Mixed (11)	Recreational (12)
daylight	0.3472***	0.2773***	0.1069***	0.2672***	0.3666***	0.4776***
	(0.0160)	(0.0437)	(0.0361)	(0.0198)	(0.0679)	(0.0785)
twilight	0.1263***	0.0539**	-0.0322*	0.2261***	0.2447***	0.0436
	(0.0099)	(0.0207)	(0.0187)	(0.0155)	(0.0621)	(0.0631)
evening_rush_daylight	0.5337***					
	(0.1695)					
morning_rush_daylight				-0.2057		
				(0.2163)		
temperature	0.0170***	0.0098***	-0.0025	0.0529***	0.0459***	0.0386***
	(0.0014)	(0.0022)	(0.0018)	(0.0024)	(0.0049)	(0.0072)
temperature^2	0.0005***	0.0009***	0.0015***	-0.0007***	-0.0002	0.0006**
	(6.05×10^{-5})	(0.0001)	(0.0002)	(7.55×10^{-5})	(0.0002)	(0.0003)
light_drizzle	-0.1104***	-0.0838***	-0.0645***	-0.1859***	-0.1832***	-0.1517***
	(0.0055)	(0.0118)	(0.0127)	(0.0065)	(0.0204)	(0.0215)
strong_drizzle	-0.1536***	-0.1446***	-0.0616***	-0.2100***	-0.2156***	-0.1441***
	(0.0080)	(0.0138)	(0.0147)	(0.0085)	(0.0236)	(0.0268)
light_rain	-0.1748***	-0.1334***	-0.0430***	-0.2408***	-0.2630***	-0.1925***
	(0.0132)	(0.0282)	(0.0120)	(0.0097)	(0.0291)	(0.0284)
moderate_rain	-0.2806***	-0.1724***	-0.0828***	-0.2677***	-0.2574***	-0.1430***
	(0.0157)	(0.0383)	(0.0218)	(0.0111)	(0.0317)	(0.0333)
strong_rain	-0.3526***	-0.2475***	-0.1767***	-0.2585***	-0.2029***	-0.1259**
	(0.0388)	(0.0576)	(0.0339)	(0.0278)	(0.0612)	(0.0496)
heavy_rain	-0.2623**	-0.2714**	-0.1167*	-0.2026***	-0.1759*	-0.0347
	(0.1118)	(0.1080)	(0.0640)	(0.0265)	(0.0861)	(0.0620)
precipitation_lag	-0.1708***	-0.1454***	-0.1091***	-0.3259***	-0.3255***	-0.2620***
	(0.0074)	(0.0104)	(0.0180)	(0.0091)	(0.0268)	(0.0309)
cloudiness	-0.0087***	-0.0080***	-0.0086***	-0.0198***	-0.0198***	-0.0316***
	(0.0005)	(0.0009)	(0.0018)	(0.0011)	(0.0026)	(0.0042)
windspeed	-0.0127***	-0.0104***	-0.0078***	-0.0289***	-0.0264***	-0.0285***
	(0.0012)	(0.0033)	(0.0019)	(0.0014)	(0.0040)	(0.0048)
general_holidays	-0.8333***	-0.6248***	-0.1581***	-0.6131***	-0.4123***	0.1727***
	(0.0265)	(0.0525)	(0.0401)	(0.0218)	(0.0615)	(0.0248)
school_holidays	-0.1527***	-0.1015***	-0.0456***	-0.1900***	-0.1053***	-0.0280*
	(0.0058)	(0.0056)	(0.0117)	(0.0094)	(0.0080)	(0.0161)
semester_break	-0.0151**	0.0051	-0.0012	-0.0456***	-0.0209	0.0937***
	(0.0061)	(0.0169)	(0.0064)	(0.0095)	(0.0279)	(0.0331)
Station FE	Yes	Yes	Yes	Yes	Yes	Yes
Weekday FE	Yes	Yes	Yes	Yes	Yes	Yes
Hour FE	Yes	Yes	Yes	Yes	Yes	Yes
Week FE	Yes	Yes	Yes	Yes	Yes	Yes
Year FE	Yes	Yes	Yes	Yes	Yes	Yes
Observations	2,017,080	465,204	556,247	2,016,511	465,545	556,813
R^2	0.72331	0.65864	0.45146	0.83501	0.79500	0.64985

Standard errors are clustered by counting station.
The variable *counts_adj* corresponds to the actual *counts* variable, but zero counts are now changed to 1 in order to include them in the log-linear regression model. This has to be done for 21 % of the observations.
Significance Codes: ***: 0.01, **: 0.05, *: 0.1.

Table S3: Sensitivity Analysis (Differentiated Negative Binomial Regression Model)

Dependent Variable:	counts					
	Morning Hours (2:00–9:59)			Evening Hours (15:00-22:59)		
	Utilitarian	Mixed	Recreational	Utilitarian	Mixed	Recreational
	(13)	(14)	(15)	(16)	(17)	(18)
daylight_spring	0.2701***	0.2341***	0.3215***	0.2066***	0.3045***	1.006***
	(0.0282)	(0.0511)	(0.0739)	(0.0185)	(0.0598)	(0.0690)
daylight_summer	0.1998***	0.1606***	0.3377***	0.1505***	0.2568***	0.9023***
	(0.0315)	(0.0491)	(0.0625)	(0.0178)	(0.0539)	(0.0918)
daylight_autumn	0.3525***	0.1835***	0.0310	0.2668***	0.3617***	1.066***
	(0.0249)	(0.0387)	(0.0819)	(0.0184)	(0.0737)	(0.0621)
daylight_winter	0.3057***	0.2062***	0.0582	0.2370***	0.3316***	0.9927***
	(0.0250)	(0.0423)	(0.0916)	(0.0198)	(0.0858)	(0.0870)
daylight_rainy	-0.1439***	-0.1969***	-0.3479***	-0.0853***	-0.1759***	-0.2510***
	(0.0140)	(0.0296)	(0.0309)	(0.0114)	(0.0325)	(0.0274)
daylight_clouded	0.0237**	0.0450	-0.0086	-0.0047	0.0451*	-0.0275
	(0.0118)	(0.0302)	(0.0309)	(0.0093)	(0.0270)	(0.0202)
twilight_spring	0.0109	-0.0076	0.1034***	0.1824***	0.2461***	0.6381***
	(0.0143)	(0.0296)	(0.0361)	(0.0139)	(0.0502)	(0.0393)
twilight_summer	0.0471**	0.0526	0.1236***	0.2048***	0.3059***	0.7210***
	(0.0194)	(0.0377)	(0.0357)	(0.0164)	(0.0591)	(0.0547)
twilight_autumn	0.3433***	0.2322***	0.1464**	0.2211***	0.2911***	0.7554***
	(0.0266)	(0.0392)	(0.0662)	(0.0154)	(0.0592)	(0.0642)
twilight_winter	0.2097***	0.1602***	0.1488**	0.1891***	0.2752***	0.6856***
	(0.0248)	(0.0316)	(0.0679)	(0.0171)	(0.0598)	(0.0687)
twilight_rainy	-0.0319*	-0.1049***	-0.1177***	-0.0620***	-0.1403***	-0.1522***
	(0.0166)	(0.0237)	(0.0294)	(0.0090)	(0.0219)	(0.0277)
twilight_clouded	0.0254*	0.0432	0.0342	-0.0291***	-0.0310**	-0.0741***
	(0.0132)	(0.0295)	(0.0399)	(0.0060)	(0.0148)	(0.0225)
evening_rush_daylight	0.4985**					
	(0.2152)					
morning_rush_daylight				-0.2378		
				(0.2213)		
temperature	0.0256***	0.0246***	0.0346***	0.0497***	0.0517***	0.1499***
	(0.0024)	(0.0022)	(0.0049)	(0.0025)	(0.0062)	(0.0093)
temperature^2	-9.34×10^{-5}	7.64×10^{-5}	0.0002	-0.0006***	-0.0004**	-0.0026***
	(7.82×10^{-5})	(9.72×10^{-5})	(0.0002)	(7.28×10^{-5})	(0.0002)	(0.0003)
light_drizzle	-0.0913***	-0.0480*	-0.0488*	-0.1316***	-0.1081***	-0.2104***
	(0.0127)	(0.0260)	(0.0252)	(0.0071)	(0.0166)	(0.0175)
strong_drizzle	-0.1412***	-0.1259***	-0.0877**	-0.1448***	-0.1153***	-0.1997***
	(0.0142)	(0.0300)	(0.0430)	(0.0082)	(0.0147)	(0.0378)
light_rain	-0.2226***	-0.2062***	-0.1196***	-0.1674***	-0.1883***	-0.2929***
	(0.0168)	(0.0398)	(0.0373)	(0.0095)	(0.0188)	(0.0297)
moderate_rain	-0.3197***	-0.2503***	-0.2059***	-0.1738***	-0.1558***	-0.1452***
	(0.0204)	(0.0522)	(0.0681)	(0.0103)	(0.0212)	(0.0342)
strong_rain	-0.4320***	-0.3962***	-0.4889***	-0.1171***	0.0108	0.0038
	(0.0389)	(0.0747)	(0.1187)	(0.0278)	(0.0616)	(0.0599)
heavy_rain	-0.4253**	-0.7652***	0.1663	-0.0476	0.0579	0.2547**
	(0.1981)	(0.1539)	(0.8315)	(0.0296)	(0.0667)	(0.1219)
precipitation_lag	-0.2255***	-0.2635***	-0.5033***	-0.3126***	-0.3599***	-0.6926***
	(0.0106)	(0.0132)	(0.0331)	(0.0089)	(0.0292)	(0.0196)
cloudiness	-0.0142***	-0.0180***	-0.0279***	-0.0175***	-0.0254***	-0.0485***
	(0.0016)	(0.0040)	(0.0048)	(0.0013)	(0.0043)	(0.0040)
windspeed	-0.0192***	-0.0229***	-0.0279***	-0.0263***	-0.0291***	-0.0571***
	(0.0015)	(0.0041)	(0.0025)	(0.0012)	(0.0044)	(0.0055)
general_holidays	-0.8389***	-0.9514***	-0.6025***	-0.5625***	-0.3599***	0.3505***
	(0.0551)	(0.0840)	(0.0796)	(0.0245)	(0.0619)	(0.0461)
school_holidays	-0.2199***	-0.1721***	-0.2074***	-0.1775***	-0.1069***	-0.0194
	(0.0075)	(0.0104)	(0.0219)	(0.0079)	(0.0062)	(0.0261)
semester_break	-0.0212**	-0.0078	0.0531	-0.0411***	-0.0544**	0.0969*
	(0.0083)	(0.0228)	(0.0436)	(0.0091)	(0.0248)	(0.0523)
Station FE	Yes	Yes	Yes	Yes	Yes	Yes
Weekday FE	Yes	Yes	Yes	Yes	Yes	Yes
Hour FE	Yes	Yes	Yes	Yes	Yes	Yes
Week FE	Yes	Yes	Yes	Yes	Yes	Yes
Year FE	Yes	Yes	Yes	Yes	Yes	Yes
Observations	2,016,916	465,184	556,223	2,016,383	465,529	556,813
Squared Correlation	0.71657	0.73188	0.60080	0.81181	0.70460	0.74998

Standard errors are clustered by counting station.
Significance Codes: ***: 0.01, **: 0.05, *: 0.1.
